# Circulating neurofilament is linked with morbid obesity, renal function, and brain density

**DOI:** 10.1038/s41598-022-11557-2

**Published:** 2022-05-12

**Authors:** Eleni Rebelos, Eero Rissanen, Marco Bucci, Olli Jääskeläinen, Miikka-Juhani Honka, Lauri Nummenmaa, Diego Moriconi, Sanna Laurila, Paulina Salminen, Sanna-Kaisa Herukka, Tarun Singhal, Pirjo Nuutila

**Affiliations:** 1 Turku PET Centre, University of Turku, Turku, Finland; 2 CNR, Pisa, Italy; 3 PET Imaging Program in Neurologic Diseases, Singhal Lab, Ann Romney Center for Neurologic Diseases, Brigham and Women’s Hospital and Harvard Medical School, Boston, MA USA; 4 Division of Clinical Geriatrics, Department of Neurobiology, Care Sciences and Society, Center for Alzheimer Research, Karolinska Institutet, Stockholm, Sweden; 5 Turku PET Centre, Åbo Akademi University, Turku, Finland; 6 Institute of Clinical Medicine-Neurology, Faculty of Health Sciences, University of Eastern Finland, Kuopio, Finland; 7 Department of Psychology, University of Turku, Turku, Finland; 8 Department of Surgical, Medical, Molecular Pathology and Critical Care Medicine, University of Pisa, Pisa, Italy; 9 Division of Digestive Surgery and Urology, Turku University Hospital, Turku, Finland; 10 Department of Surgery, University of Turku, Turku, Finland; 11 Neurocenter, Kuopio University Hospital, Kuopio, Finland; 12 Department of Neurology, Brigham Multiple Sclerosis Center, Ann Romney Center for Neurologic Diseases, Brigham and Women’s Hospital, Harvard Medical School, Boston, MA USA; 13 Department of Endocrinology, Turku University Hospital, Turku, Finland

**Keywords:** Neuroscience, Medical research, Neurology

## Abstract

Neurofilament light chain (NfL) is a novel biomarker reflecting neuroaxonal damage and associates with brain atrophy, and glial fibrillary acidic protein (GFAP) is a marker of astrocytic activation, associated with several neurodegenerative diseases. Since obesity is associated with increased risk for several neurodegenerative disorders, we hypothesized that circulating NfL and GFAP levels could reflect neuronal damage in obese patients. 28 morbidly obese and 18 lean subjects were studied with voxel based morphometry (VBM) MRI to assess gray and white matter densities. Serum NfL and GFAP levels were determined with single-molecule array. Obese subjects were re-studied 6 months after bariatric surgery. Morbidly obese subjects had lower absolute concentrations of circulating NfL and GFAP compared to lean individuals. Following bariatric surgery-induced weight loss, both these levels increased. Both at baseline and after weight loss, circulating NfL and GFAP values correlated inversely with eGFR. Cross-sectionally, circulating NfL levels correlated inversely with gray matter (GM) density, and this association remained significant also when accounting for age and total eGFR. GFAP values did not correlate with GM density. Our data suggest that when determining circulating NfL and GFAP levels, eGFR should also be measured since renal function can affect these measurements. Despite the potential confounding effect of renal function on NfL measurement, NfL correlated inversely with gray matter density in this group of subjects with no identified neurological disorders, suggesting that circulating NfL level may be a feasible biomarker of cerebral function even in apparently neurologically healthy subjects.

## Introduction

Obesity and obesity-related impairments in glucose homeostasis present as risk factors for several neurodegenerative disorders, including Alzheimer’s disease (AD)^1,2^. With the ever increasing obesity pandemic^3,4^, the risk for increasing prevalence of neurodegenerative disorders is imminent.

Magnetic resonance imaging (MRI) is the current standard to quantitate brain atrophy as a macroscopic reflection of the neuroaxonal damage due to disease or aging. Obesity has also been shown to associate with decreased brain volume^5^, and importantly, these structural changes in obesity have been suggested to reflect early stages of neuronal damage, possibly leading to irreversible neurodegeneration in obesity^6^. However, MRI is a relatively expensive neuroimaging tool and not easily accessible to a large number of patients. Less expensive circulating biomarkers would be essential as a screening tool for brain atrophy.

A new potential biomarker of neurodegeneration is neurofilament light chain (NfL), which is one of the scaffold proteins specific for neuronal cytoskeleton^7^. Upon neuro-axonal injury, NfL is released into extracellular space^8^, and elevated NfL levels in the cerebrospinal fluid and peripheral blood have been shown to indicate axonal injury in several neurologic diseases^9,10^. Glial fibrillary acidic protein (GFAP) is an intracellular protein of the astrocytic cytoskeleton, and increases in central nervous system (CNS) GFAP and circulating GFAP levels have been described in the context of traumatic brain injury (TBI)^11^, in the context of neurodegeneration in AD^12^ as well as in chronic neuroinflammatory conditions such as multiple sclerosis (MS)^13^.

We have previously shown that obesity is linked to decreased brain density, and that weight loss following bariatric surgery leads to global increase in white matter (WM), and increase in gray matter (GM) density in occipital and inferior temporal regions^14^. In this study, we further evaluated the effect of obesity and weight loss following bariatric surgery on the circulating levels of NfL and GFAP, on the same subjects reported by Tuulari et al*.*^14^. Moreover, we assessed whether circulating NfL and GFAP correlate with brain density.

## Materials and methods

### Participants and study design

The initial dataset comprised of 28 morbidly obese and 19 lean controls. All study participants were free of neurological disease (e.g., large vessel stroke, seizure disorder, Parkinson’s or Alzheimer’s disease, clinically significant traumatic brain injury, multiple sclerosis, or previous brain infection/meningitis); and none of them had any major psychiatric illness (e.g., schizophrenia, bipolar disorder), nor reported substance abuse. Subjects were also eligible to undergo magnetic resonance imaging. In one lean subject, the NfL level was clearly in the abnormal range (NfL value 37.8 (ng/L)). After checking her medical records, we identified that the subject had undergone surgery for a large lumbar disc prolapse causing stenosis 9 months before her participation in the study. As increases in circulating NfL levels may be associated with herniated and spondolytic intervertebral discs^15^, we excluded this subject from further analysis, leaving 28 morbidly obese and 18 lean controls in the dataset. Patients with obesity were studied before and six months after bariatric surgery. Lean subjects were studied once. Inclusion and exclusion criteria and the surgical techniques have been previously described in detail^16^. Altogether, 8 subjects from the morbidly obese group had pre-diabetic conditions (impaired fasting glucose (IFG) or impaired glucose tolerance (IGT)) and 11 had type 2 diabetes mellitus (T2D) as defined by ADA criteria^17^. All lean subjects had normal glucose tolerance (NGT). The study protocol was approved by the Ethics Committee of the Hospital District of Southwest Finland, all research was performed in accordance with relevant guidelines/regulations and all subjects gave their written informed consent before participating in the study (NCT00793143 and NCT01373892).

### Neurofilament and GFAP measurement

The analyses of serum NfL and GFAP were performed by the Biomarkers for Neurodegenerative Disorders research group in University of Eastern Finland (Kuopio, Finland; https://www3.uef.fi/fi/web/neuro/biomarkers) using the single-molecule array (SIMOA) platform (Quanterix Corporation, Billerica, MA, USA) (“Advantage” and “Discovery” kits for NfL and GFAP, respectively). In 8/46 subjects the GFAP assessment failed due to high replicate variance (coefficient of variation (CV) > 20%), and thus, GFAP data were available for evaluation in 38 subjects. Mean CV of the sample replicates was 5.2% for NfL and 6.9% for GFAP. Variance of control material was within acceptable limits based on internal quality control protocol (± 3SD).

### Analytical methods

Plasma glucose was measured in the laboratory of Turku PET Centre in duplicate using the glucose oxidase technique (Analox GM7 or GM9, Analox Instruments Ltd., London, UK). Glycosylated hemoglobin (HbA_1c_) was measured with ion-exchange high performance liquid chromatography (Variant II Haemoglobin A_1c_, Bio-Rad Laboratories, CA, USA). Plasma insulin was determined by time-resolved immunofluorometric assay (AutoDELFIA, Perkin Elmer Inc, Wallac Oy, Turku, Finland). Serum FFA were measured with a photometric enzymatic assay (Wako Chemicals GmbH, Neuss, Germany) on Modular P800 automatic analyzer (Roche Diagnostics, Mannheim, Germany). Serum high-sensitivity C-reactive protein (hs-CRP) was analysed with the sandwich immunoassay method using an Innotrac Aio1 immunoanalyzer (Innotrac Diagnostics, Turku Finland). Serum adipokines were analysed in duplicate by using Milliplex Human Serum Adipokine (Panel A) kit [cat.no HADK1-61K-A] (MilliporeSigma, Burlington, MA, USA) containing interleukin-6 (IL-6), interleukin-8 (IL-8), tumor necrosis factor alpha (TNFα), and leptin.

### MRI studies

MR imaging was performed with Philips Gyroscan Intera 1.5 T CV Nova Dual scanner at Turku PET Centre. 3D anatomical images with isotropic 1 mm^3^ resolution were acquired using a T1-weighted sequence (TR 25 ms, TE 4.6 ms, flip angle 30, scan time 376 s, image dimensions (X,Y,Z): 256 × 256 × 330, FFE SENSE protocol).

### Voxel-based morphometry (VBM)

Prior to analysis, the image quality was checked visually, and the origo of each T1 image was set to anterior commissure. Structural images were spatially normalized and segmented using the MAGIA pipeline (https://aivo.utu.fi/magia/) which utilizes Matlab (The MathWorks Inc., Natick, MA) and the Statistical Parametric Mapping toolbox (SPM 12) (http://www.fil.ion.ucl.ac.uk/spm/). Additionally, the DARTEL algorithm (in SPM12)^18^ was applied to achieve non-linear correction for GM and WM that takes into account the changes in brain volume and minimizes possible biases via creation of multiple templates. Finally, the GM and WM images, at this stage called GM density and WM density images, were smoothed using a Gaussian kernel of 8 mm full width at half maximum (FWHM).

### Total blood volume equations

Total blood volume was estimated according to the Nadler’s formula^19^. Since this formula has been criticized for overestimating total blood volume in extreme BMI range and we had some subjects with morbid obesity, we also calculated total blood volume using the Lemmens formula^20^. These measurements of estimated total blood volume correlated positively and strongly to one another (*r* = 0.99, *p* < 0.0001).

Estimated glomerular filtration rate (eGFR) was calculated by the Chronic Kidney Disease Epidemiology Collaboration (CKD-EPI) equation^21^.

Body surface area (BSA) was estimated as previously described by Du Bois&Du Bois^22^.

### Statistical analysis

Data are presented as mean ± SD (or median [IQR] for non-normally distributed variables). Whole-brain statistical analysis was performed with SPM12 running on Matlab. Linear regressions were performed in SPM to evaluate correlations between VBM and single regressors (NFL, GFAP) while controlling for confounding factors (in the SPM contrast, the controlling variables were set to a value of 0). The statistical threshold in SPM analysis was set at a cluster level and corrected with false discovery rate (FDR) with p < 0.05. Further statistical analyses were done using JMP version 13.0 (SAS Institute, Cary, NC, USA). A *p* value < 0.05 was considered statistically significant.

## Results

### Baseline

Our population consisted predominantly of women (91%). As expected, obese subjects were more insulin resistant compared to the lean individuals and had higher circulating inflammatory marker (CRP) and higher leptin levels. Even though obese subjects had non-significantly lower total cholesterol values from the lean controls, they had significantly lower HDL cholesterol values. There were no differences in eGFR (ml/1.73 m^2^/min) between obese and lean individuals, but eGFR expressed in ml/min accounting for each subject’s BSA was significantly higher in patients with obesity. There was no difference between the two groups in terms of blood pressure and age. The baseline characteristics of the study participants are reported in Table 1.

**Table 1:** Anthropometric and biochemical characteristics of the study participants.

	Controls	Obese		*p* value
		Pre	Post	
M/W	2/16	2/26	–	0.7
NGT/IFG&IGT/T2D	19/0/0	9/8/11	22/2/4	< 0.0001
Age (years)	45 ± 10	45 ± 10	–	0.2
BMI (kg m^−2^)	23.4 [3.5]	41.2 [5.7]	29.6 [5.7]*^,#^	< 0.0001
HbA_1c_ (%)	5.6 [0.5]	5.9 [0.6]	5.6 [0.5]*	0.6
Systolic BP (mmHg)	131 ± 13	137 ± 16	128 ± 15*	0.4
Diastolic BP (mmHg)	82 ± 9	86 ± 10	80 ± 9*	0.1
Total cholesterol (mmol/L)	4.6 ± 0.9	4.2 ± 0.8	4.1 ± 0.7	0.2
LDL cholesterol (mmol/L)	2.4 ± 0.7	2.4 ± 0.7	2.2 ± 0.7	0.9
HDL cholesterol (mmol/L)	1.8 ± 0.4	1.2 ± 0.3	1.4 ± 0.3*	< 0.0001
Triglycerides (mmol/L)	0.7 ± 0.3	1.3 ± 0.5	1.1 ± 0.5*	< 0.0001
Smoking (yes/no)	0/19	6/22	2/22*	0.03
Menopause (yes/no)	10/9	12/16	12/16	0.5
CRP (mg/L)	0.6 [1.0]	3.1 [4.0]	1.1 [1.4]	< 0.0001
IL-6 (ng/L)	1.8 [2.0]	2.5 [1.3]	1.9 [0.3]	0.2
IL-8 (ng/L)	4.3 [3.1]	5.1 [3.7]	8.1 [5.3]	0.1
Leptin (μg/L)	5.6 [9.6]	48.8 [25.9]	25.5 [15.5]*^,#^	< 0.0001
TNFα (ng/L)	4.0 [3.9]	4.7 [3.1]	7.5 [2.9]	0.2
HOMA-IR	1.3 [1.8]	3.2 [4.1]	1.5 [1.2]	< 0.0001
Total intracranial volume (L)	1.5 [0.2]	1.4 [0.1]	1.4 [0.1]	0.04
Serum creatinine (μmol/L)	61 ± 11	61 ± 7	62 ± 11	0.7
eGFR (ml/1.73 m^2^/min)	102 ± 10	104 ± 12	103 ± 15	0.9
Total eGFR (ml/min)	103 ± 10	131 ± 21	117 ± 20*	< 0.0001
Neurofilament (ng/L)	10.7 [5.0]	6.7 [4.0]	9.5 [5.1]*	0.002
GFAP (ng/L)	208 [111]	135 [72]	169 [112]*	0.01

Entries are mean ± SD, or median [interquartile range], as appropriate. *p* value for the comparison between obese pre and lean individuals; *p < 0.05 for obese before and after bariatric surgery; ^#^*p* < 0.05 for the comparison obese post vs. lean individuals. *BMI* body mass index, *NGT* normal glucose tolerance, *IFG* impaired fasting glucose, *IGT* impaired glucose tolerance, *T2D* type 2 diabetes, *BP* blood pressure, *CRP* C-reactive protein, *HOMA-IR* homeostatic model assessment of insulin resistance, *eGFR* estimated glomerular filtration rate, *GFAP* glial fibrillary acidic protein.

**Figure 1:**
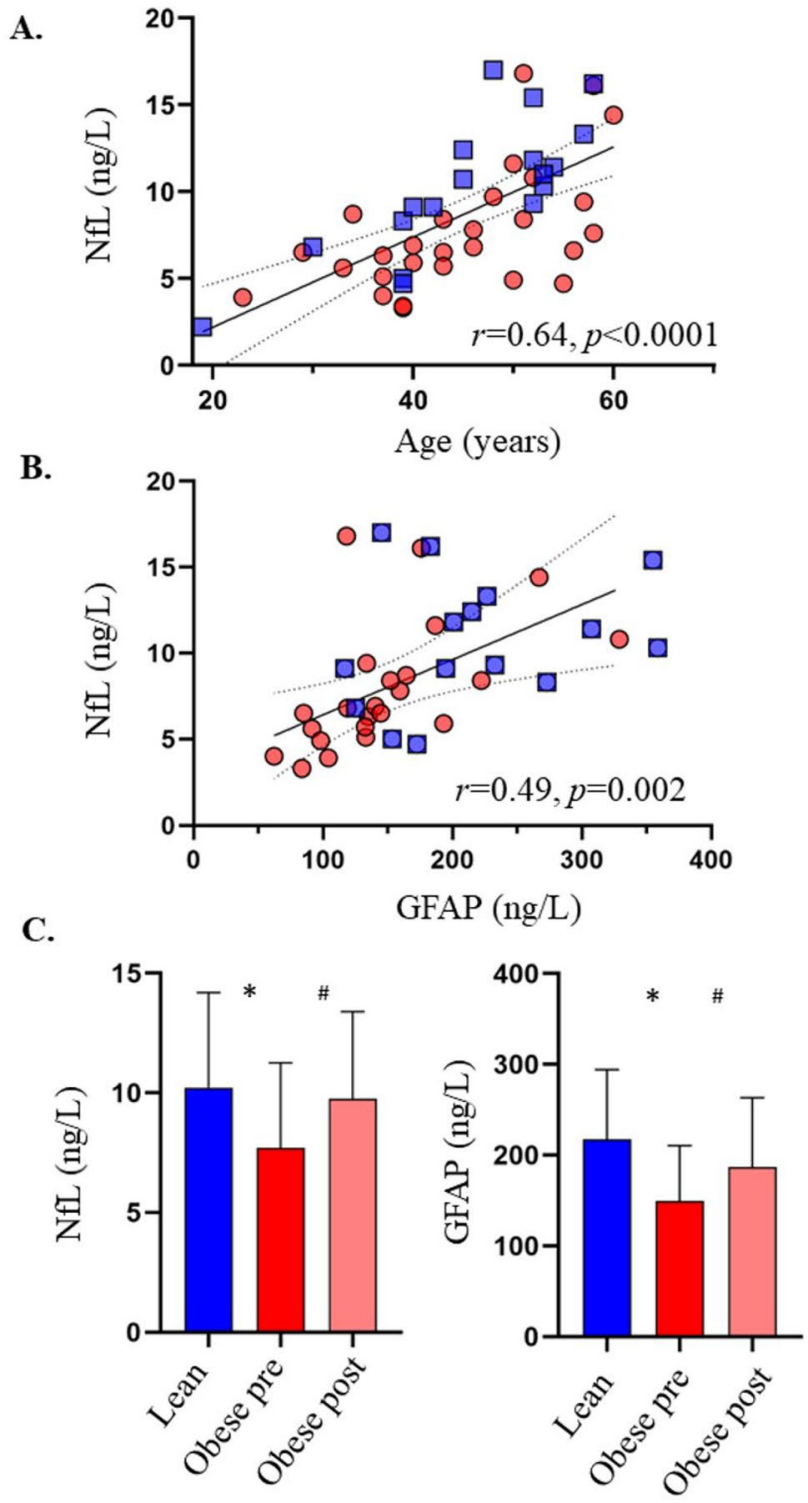
Plasma NfL levels correlated positively with age (**A**), and with GFAP concentrations (**B**). Plasma NfL and GFAP were higher in the lean as compared to the obese individuals, and increased 6-months post bariatric surgery (**C**). Red circles: subjects with obesity; blue squares: lean subjects. Light pink: obese subject post bariatric surgery. *GFAP* glial fibrillary acidic protein, *NfL* Neurofilament light chain.

**Figure 2:**
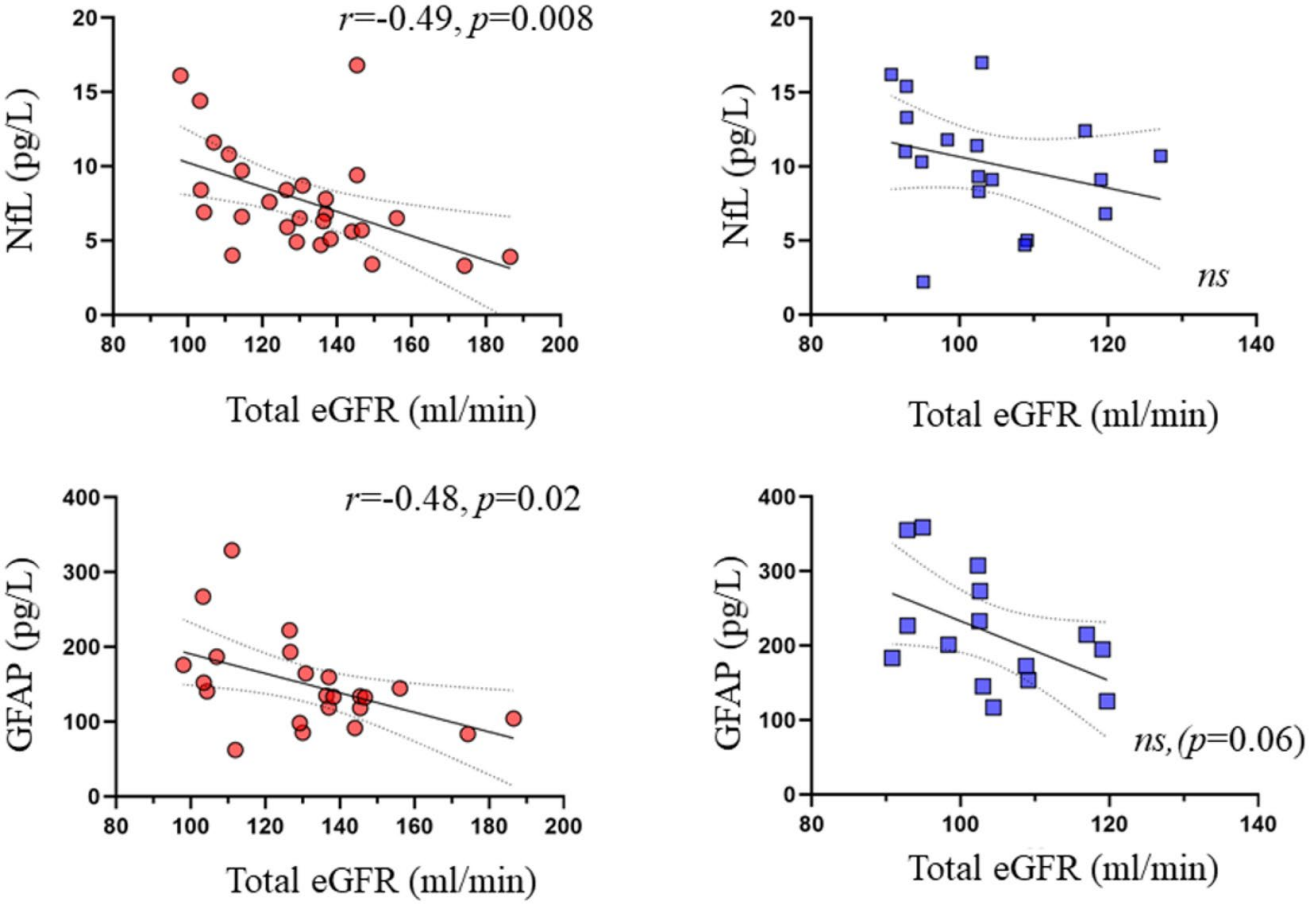
The correlation between NfL and GFAP with total eGFR was driven by the obese subjects. Red circles: subjects with obesity; blue squares: lean subjects.

In the pooled data, NfL correlated positively with age (*r* = 0.64, *p* < 0.0001), and negatively with BMI (*r* = − 0.32, *p* = 0.03) among the 46 study participants (Fig. 1). In the 38 subjects who also had circulating GFAP values measured, there was a positive correlation between NfL and GFAP (*r* = 0.49, *p* = 0.002) (Fig. 1). In the pooled data, NfL and GFAP concentrations correlated inversely with estimated glomerular filtration rate expressed per 1.73 m^2^ of body surface area (BSA) (*r* = − 0.45, *p* = 0.002, *r* = − 0.54, *p* = 0.0005, respectively). This correlation became stronger when estimated glomerular filtration rate was expressed in ml/min (*r* = − 0.50, *p* = 0.0003 and *r* = − 0.58, *p* = 0.0001, for NfL and GFAP respectively) (Fig. 2). In a multivariate analysis, age (standardized β = 0.62, t ratio 5.0, *p* < 0.0001) and interaction between age and eGFR (β = − 0.24, t ratio − 2.2, *p* = 0.033) were the best predictors for NfL concentrations, and BMI (β = − 0.30, t ratio − 2.0, *p* = 0.055) had a borderline association, whereas eGFR did not show meaningful independent contribution (β = − 0.15, t ratio − 0.8, *p* = 0.407). Total blood volume was not included in this multivariate model due to its high correlation with BMI (*r* = 0.84) and eGFR (*r* = 0.78).

Regarding GFAP, in univariate analysis age (*r* = 0.52, *p* = 0.0009), BMI (*r* = − 0.41, *p* = 0.001), eGFR (*r* = − 0.58, *p* = 0.0001), and total blood volume (*r* = − 0.32, *p* = 0.052) were all significantly related to GFAP. In multivariate analysis, only age (β = 0.36, t ratio − 2.2, *p* = 0.036) was significant predictor for GFAP, whereas interaction between age and eGFR (β = − 0.26, t ratio − 1.9, *p* = 0.068) and eGFR (β = − 0.36, t ratio − 1.6, *p* = 0.117) had borderline associations, and BMI (β = − 0.17, t ratio − 0.9, *p* = 0.369) did not show meaningful independent contribution.

### Following bariatric surgery

**Figure 3:**
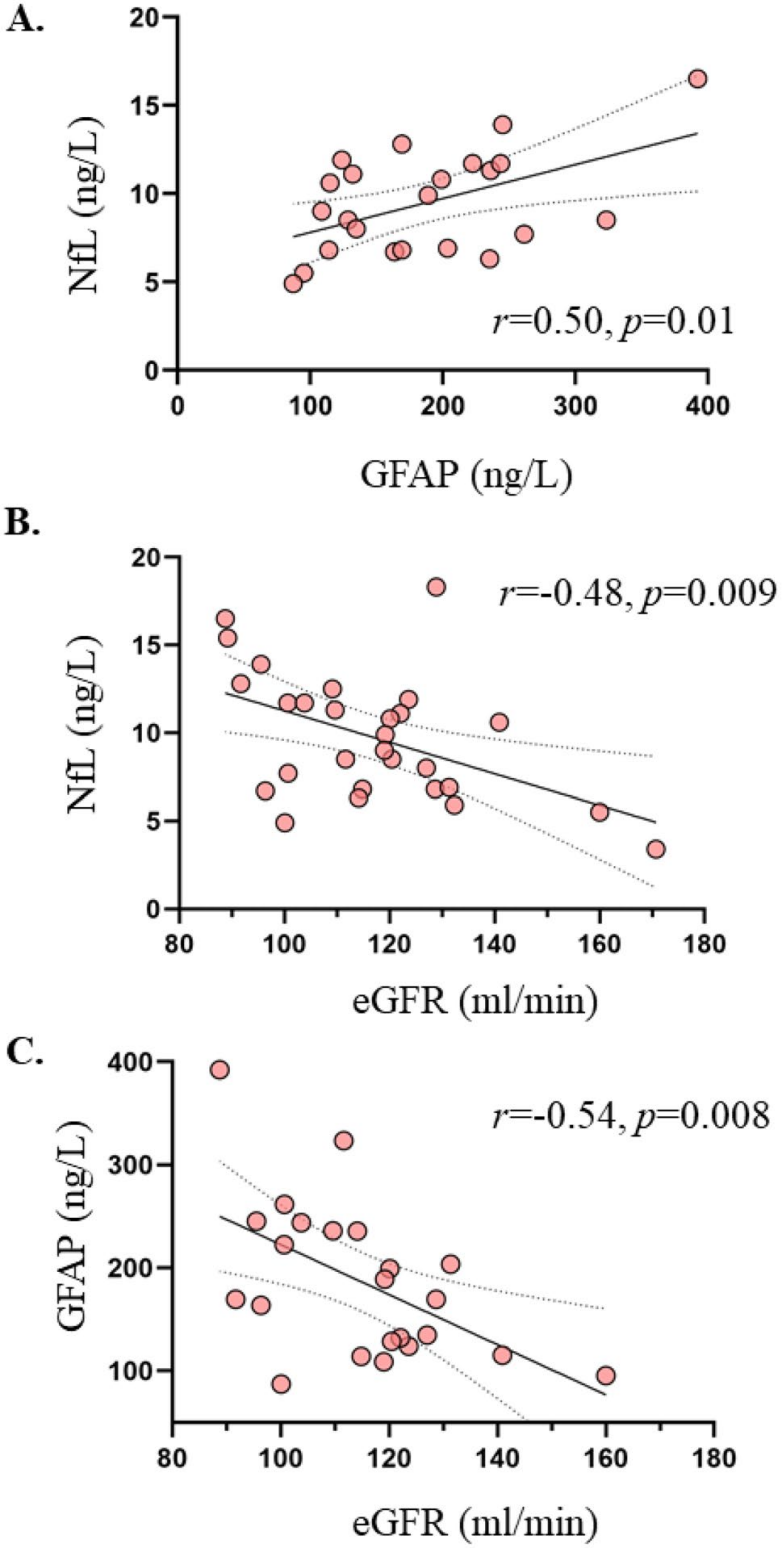
6-months after bariatric surgery circulating NfL and GFAP levels were directly related to each other (**A**), and correlated inversely with total eGFR (**B**, **C**). *GFAP* glial fibrillary acidic protein, *NfL* neurofilament light chain, *eGFR* estimated glomerular filtration rate.

Patients undergoing bariatric surgery achieved significant weight loss with an average weight loss of approximately 10 units of BMI at six months after surgery. Insulin sensitivity, glucose tolerance status, and markers of systemic inflammation were also improved (Table 1). Circulating NfL and GFAP values were increased, and total eGFR decreased after surgery, and at the postoperative assessment, there was no difference with the plasma NfL and GFAP values of the lean subjects, and they still correlated positively with each other (*r* = 0.50, *p* = 0.01) (Table 1, Figs. 1, 3). Furthermore, circulating NfL and GFAP values were still negatively correlating with estimated glomerular filtration rate (Fig. 3).

### NfL and VBM

**Figure 4:**
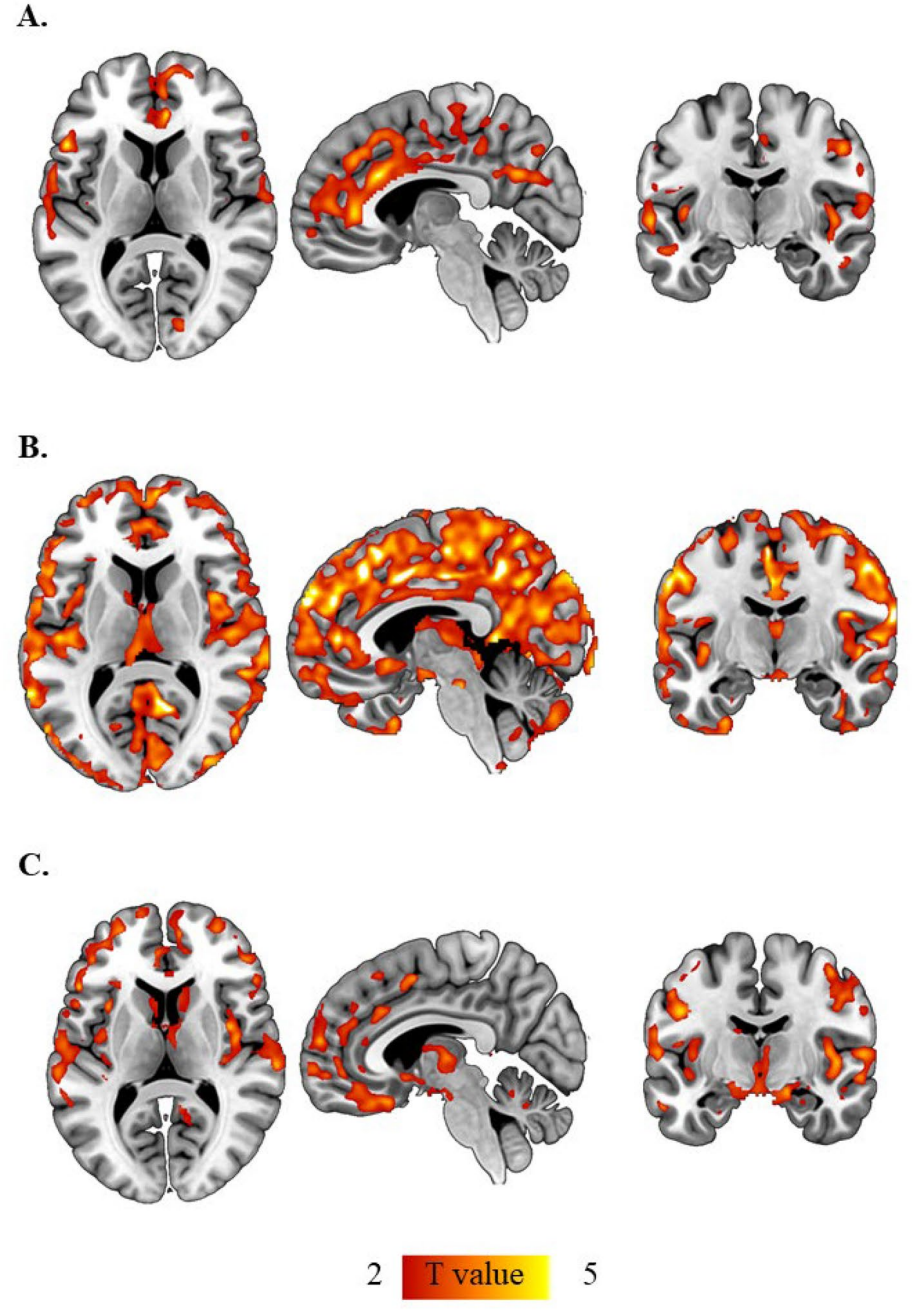
Plasma Neurofilament light chain correlated inversely with gray matter density in the whole dataset (**A**). This correlation was more widespread in the lean (**B**), compared to the obese (**C**) individuals. Statistical parametric mapping results (*p* < 0.05, false discovery rate (FDR) corrected; extent threshold k: 9937 (**A**), extent threshold k: 1120 (**B**); Extent threshold k: 7614 (**C**), X: 5, Y: − 10, Z: 9.5). Images were created using Mango (Multi-image Analysis GUI) software, version 4.1 (http://rii.uthscsa.edu/mango/).

In the whole dataset, circulating NfL levels correlated inversely with GM density in hippocampi, cingulate gyrus and in wide-spread and partially scattered areas in frontal, parietal and occipital lobes. When dividing the population into obese and lean individuals, lean subjects showed widespread correlations between cortical GM and medial thalamic densities and NfL, whereas in the obese subjects the correlation was restricted mainly to the frontotemporal cortices, head of caudate, and hippocampi (Fig. 4). In both groups, the correlations between GM density and NfL levels remained significant after accounting for age and total blood volume, or after accounting for age and glomerular filtration rate (Supplementary Fig. 1A,B). No correlation was found between white matter density and circulating NfL levels. GFAP did not correlate with gray or white matter densities in the whole dataset, or when dividing the population in the two subgroups.

At 6 months after bariatric surgery, circulating NfL levels were still negatively associated with GM density (Supplementary Fig. 1C).

## Discussion

In a sample of young and middle-aged individuals, across a wide range of BMI and insulin sensitivity with no clinical diagnosis of neurologic disorders, circulating plasma NfL levels correlated inversely with gray matter density in hippocampi, cingulate gyrus, and in frontal, parietal, and occipital lobe regions. In contrast to our hypothesis plasma NfL and GFAP concentrations were lower in patients with obesity compared to lean subjects, but at six months after bariatric surgery with significant weight loss, NfL and GFAP levels were increased to the same level as in lean controls. While circulating NfL and GFAP concentrations were directly related both at pre- and post-surgery, we found a significant inverse correlation between both biomarkers and estimated glomerular filtration rate both at baseline and following bariatric surgery.

NfL has been proposed as a useful biomarker for several neurologic diseases since increased NfL concentrations in the cerebrospinal fluid or blood indicate axonal injury^9,10^. Since obesity is linked to decreased brain density^14^, and it represents a risk factor for neurodegeneration^23^, our hypothesis was that morbidly obese subjects would have increased circulating NfL values, and that following weight loss NfL values would have been decreased. Similarly, GFAP is expressed in activated astrocytes^24^, and is a marker for astrogliosis in the CNS^24^, and thus, we would have expected higher circulating GFAP levels in the obese^25–28^. Contrary to our hypotheses, we found higher circulating levels of both these two biomarkers in the lean subjects compared to the obese individuals.

Indeed, in a recent large study, Manouchehrinia and colleagues have shown that BMI and blood volume correlate negatively with circulating plasma NfL levels, whereas no such association was found between CSF NfL levels and BMI^29^. Importantly, one key feature in obesity is glomerular hyperfiltration^30^ which in the long run constitutes the basis for obesity-induced chronic renal failure^31^. Our data showed that in patients with obesity, both biomarkers correlated inversely with eGFR both at baseline and after bariatric surgery, suggesting that the degree of renal function may affect their circulating levels. This finding is in agreement with the recent report by Akamine et al., where it was also shown that circulating plasma NfL levels correlated positively with serum creatinine and negatively with estimated glomerular filtration rate (eGFR) in healthy controls and patients with T2D^32^. Further, in the multivariate regression analyses, age and eGFR interaction term was a significant predictor for NfL and had a similar trend for GFAP suggesting that the effect of age on circulating NfL and GFAP concentrations are partially mediated by eGFR further highlighting the finding by Akamine et al.

Obesity is a risk factor for microalbuminuria and proteinuria^33^. Pathophysiological mechanisms include alteration of the tubulo-glomerular feedback, endothelial dysfunction linked to chronic microinflammation^33^, and reduced reabsorption in the proximal tubules^34^. The molecular weights of NfL (61.5 kDa^35^) and GFAP (50 kDa^36^) are similar to that of albumin (66.5 kDa), and thus, obesity could also lead to relatively higher loss of these proteins in urine, explaining the decreased circulating levels of NfL and GFAP in the obese.

In further support of this, the association between NfL (or GFAP) and eGFR was driven by the subjects with obesity, whereas no such correlation was found in the lean individuals. Unfortunately, urine samples were not collected in the current dataset, and thus, future research is warranted to establish whether NfL and GFAP are cleared by the kidneys, and whether their excretion correlates with the level of albuminuria. More specifically, a study assessing contemporaneously CSF, plasma, and urine concentrations of NfL and GFAP would be most informative. This could also have implications on attempts to establish diagnostic reference values as well as on interpreting longitudinal changes and treatment responses in neurologic diseases.

Despite a significant interaction with glomerular filtration rate, we found an inverse correlation between NfL levels and gray matter density. This inverse correlation may reflect an association between neuroaxonal damage and decreased neuronal density in obesity. However, even more wide-spread correlations were observed among healthy controls, which warrants further studies. Since our dataset comprised of apparently neurologically healthy individuals who were discordant only in terms of BMI, we hypothesize that the stronger correlations of GM density with NfL levels in lean subjects is suggestive of the more accurate measurement of NfL in these subjects. Circulating NfL values may be less representative in the obese because of higher filtration and excretion of the protein in subjects with obesity, thus masking the correlation with GM density to some extent. In line with our findings, another recent study has also demonstrated that higher circulating NfL levels associate with lower total brain volumes and higher age^37^. The association between decreased brain density and higher circulating NfL in our study may also represent a preceding stage before the development of more gross atrophy, which may be driven by normal aging as well as subclinical comorbidities^37^.

On the contrary, we did not find any association between circulating GFAP levels and GM density. However, one salient finding of the present study is the close correlation between NfL and GFAP concentrations both in the pooled data in obese and lean individuals, and in obese subjects six months following bariatric surgery induced weight loss. This finding is in accordance with previous studies showing the close correlation of the two circulating proteins in patients with MS^38^ and in healthy controls^39,40^. What is of particular interest in the present study, is that this close correlation was also found in neurologically healthy subjects. From a pathophysiologic standpoint, in CNS injury astrocytes get activated and proliferate expressing the GFAP in an attempt to limit the insulting process^41^. However, astrocytes may also secrete proinflammatory mediators and thus eventually contribute to the CNS damage^42^. Interestingly, diet-induced obesity and high-fat diet may be associated with hypothalamic inflammation and concomitant astrogliosis and GFAP overexpression^43^, but in order to detect subtle morphometric changes in hypothalamus and their associations with circulating GFAP levels, larger sample sizes than in our current study may be needed.

There are some important limitations in this study. First, our sample comprised mainly of women, and thus, extrapolation of our findings to men should be done with caution. Even though our studied population comprised of apparently neurologically healthy individuals, cognitive function was not formally evaluated. Thus, in future research it would be interesting to study whether circulating NfL levels correlate with cognitive function using standardized and validated assessment methods in subjects with morbid obesity.

In conclusion, our data suggest that decreased circulating NfL and GFAP levels found in patients with obesity may be based on their enhanced glomerular filtration rate. However, circulating NfL correlated inversely with gray matter densities in this dataset comprised of apparently neurologically healthy subjects, suggesting that this circulating biomarker may be useful even in detecting associations with subtle alterations in brain density. Based on our data, we suggest considering other possible confounders, such as BMI and GFR, when determining plasma NfL and GFAP.

## Supplementary Information

Supplementary Figure S1.

## Abbreviations

AD: Alzheimer’s disease
BMI: Body mass index
eGFR: Estimated glomerular filtration rate
GFAP: Glial fibrillary acidic protein
GM: Gray matter
IFG: Impaired fasting glucose
IGT: Impaired glucose tolerance
MRI: Magnetic resonance imaging
NFL: Neurofilament light chain
NGT: Normal glucose tolerance
Simoa: Single-molecule array
SPM: Statistical parametric mapping
T2D: Type 2 diabetes
VBM: Voxel-based morphometry
WM: White matter
