# Supplementary material for: Circulating neurofilament is linked with morbid obesity, renal function, and brain density
Source: Sci Rep. 2022 May 12;12:7841. doi: 10.1038/s41598-022-11557-2 (PMC9098484; doi:10.1038/s41598-022-11557-2)

SUPPLEMENTARY MATERIAL

**Circulating neurofilament is linked with morbid obesity, renal function, and brain density**

Eleni Rebelos^1,2^*, Eero Rissanen^1,3^*, Marco Bucci^1,4,5^, Olli Jääskeläinen^6^, Miikka-Juhani Honka^1^, Lauri Nummenmaa^1,7^, Diego Moriconi^8^, Sanna Laurila^1^, Paulina Salminen^9,10^, Sanna-Kaisa Herukka^6,11^, Tarun Singhal^3,12^, Pirjo Nuutila^1,13^

Short title: neurofilament and GFAP and obesity

^1^ Turku PET Centre, University of Turku, Turku, Finland

^2^ CNR, Pisa, Italy

^3^ PET Imaging Program in Neurologic Diseases, Singhal Lab, Ann Romney Center for Neurologic Diseases, Brigham and Women's Hospital and Harvard Medical School, Boston, MA, USA

^4^ Division of Clinical Geriatrics, Center for Alzheimer Research, Department of Neurobiology, Care Sciences and Society, Karolinska Institutet, Stockholm, Sweden

^5^ Turku PET Centre, Åbo Akademi University, Turku, Finland

^6^ Institute of Clinical Medicine – Neurology, Faculty of Health Sciences, University of Eastern Finland, Kuopio, Finland

^7^ Department of Psychology University of Turku, Turku, Finland

^8^ Department of Surgical, Medical, Molecular Pathology and Critical Care Medicine
University of Pisa

^9^ Division of Digestive Surgery and Urology, Turku University Hospital, Turku, Finland

^10^ Department of Surgery, University of Turku, Turku, Finland

^11^ Neurocenter, Kuopio University Hospital, Kuopio, Finland

^12^ Brigham Multiple Sclerosis Center, Ann Romney Center for Neurologic Diseases, Department of Neurology, Brigham and Women's Hospital, Harvard Medical School, Boston, MA.

^13^ Department of Endocrinology, Turku University Hospital, Turku, Finland

**Supplementary Figure 1:**

The association between plasma NfL and gray matter density remained significant after accounting for eGFR in the obese (**A**) and lean subjects (**B**), respectively. Also following bariatric surgery plasma NfL correlated inversely with gray matter density (**C**). Statistical parametric mapping results (*p*<0.05, FDR corrected). Images were created using Mango (Multi-image Analysis GUI) software, version 4.1 (http://rii.uthscsa.edu/mango/).


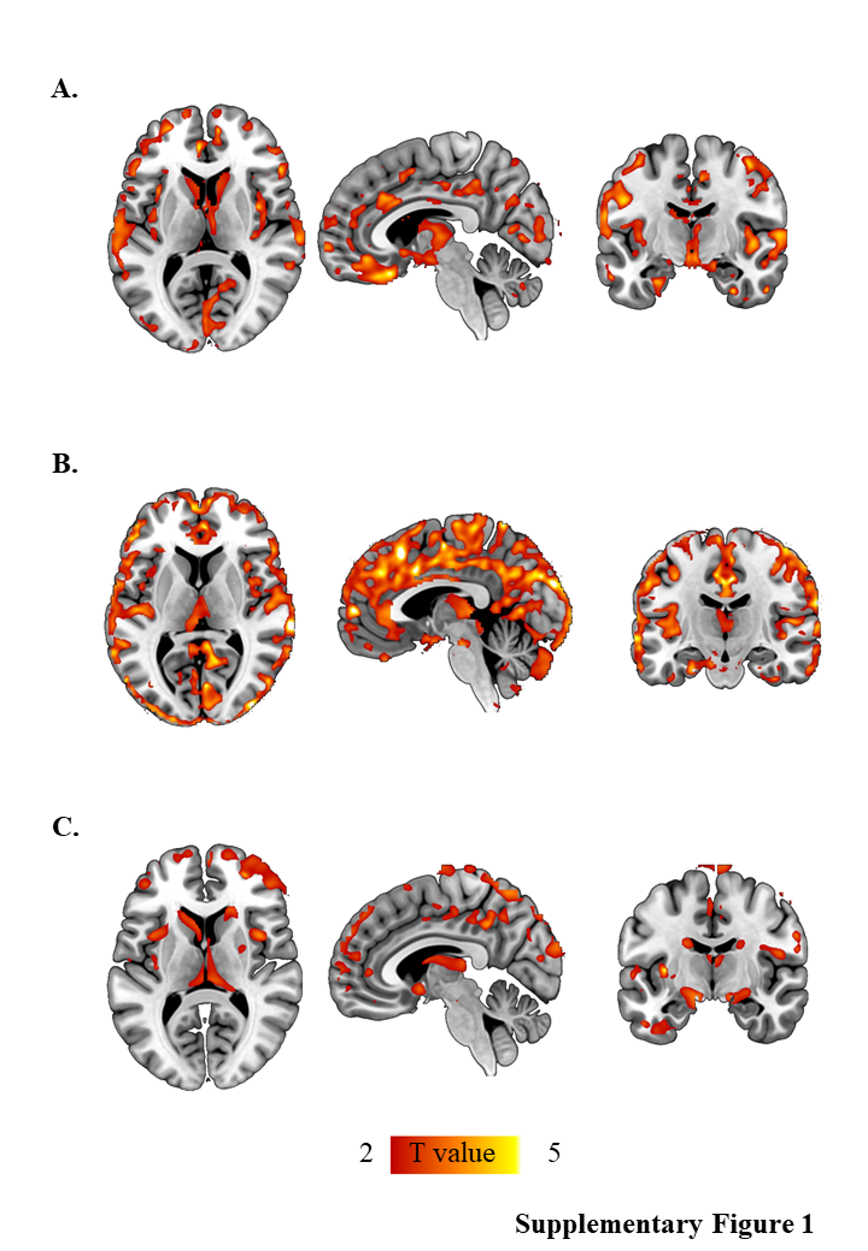

Supplement: Supplementary file 1 — Supplementary Figure S1. [file 41598_2022_11557_MOESM1_ESM.docx]
